# CRISPR-mediated activation of endogenous BST-2/tetherin expression inhibits wild-type HIV-1 production

**DOI:** 10.1038/s41598-019-40003-z

**Published:** 2019-02-28

**Authors:** Yanzhao Zhang, Seiya Ozono, Weitong Yao, Minoru Tobiume, Shoji Yamaoka, Satoshi Kishigami, Hideaki Fujita, Kenzo Tokunaga

**Affiliations:** 1 Department of Pathology, National Institute of Infectious Diseases, Tokyo, 162-8640 Japan; 2 Faculty of Life and Environmental Sciences, University of Yamanashi, Yamanashi, 400-8510 Japan; 3 Department of Molecular Virology, Tokyo Medical and Dental University, Tokyo, 113-8519 Japan; 4 Faculty of Pharmaceutical Sciences, Nagasaki International University, Nagasaki, 859-3298 Japan

## Abstract

The CRISPR technology not only can knock out target genes by using the RNA-guided Cas9 nuclease but also can activate their expression when a nuclease-deficient Cas9 (dCas9) is employed. Using the latter function, we here show the effect of the CRISPR-mediated pinpoint activation of endogenous expression of BST-2 (also known as tetherin), a virus restriction factor with a broad antiviral spectrum. Single-guide RNA (sgRNA) sequences targeting the BST-2 promoter were selected by promoter assays. Potential sgRNAs and dCas9 fused to the VP64 transactivation domain, along with an accessory transcriptional activator complex, were introduced into cells by lentiviral transduction. Increased expression of *BST-2* mRNA in transduced cells was confirmed by real-time RT-PCR. Cells in which BST-2 expression was highly enhanced showed the effective inhibition of HIV-1 production and replication even in the presence of the viral antagonist Vpu against BST-2. These findings confirm that the physiological stoichiometry between host restriction factors and viral antagonists may determine the outcome of the battle with viruses.

## Introduction

In the mid ‘90 s, the requirements of the so-called “accessory” (or “auxiliary”) HIV proteins sthat had been long known to be nonessential for viral replication in *in vitro* culture were reported to depend on the cell-types used for infection experiments^1–6^. Since 2002, these cell-type-dependent requirements of accessory proteins have been explained by the discovery of HIV restriction factors that are present in a cell-type dependent manner. These findings have indicated that HIV replication in cells expressing specific restriction factors can be achieved only by using viral accessory proteins to counteract them and evade their inhibitory activity through a one-on-one confrontation, such as Vif versus APOBEC3 proteins^7–10^, Vpu versus BST-2/tetherin (referred to hereafter as BST-2)^11,12^, Vpx versus SAMHD1^13,14^, and Nef versus SERINC5^15,16^.

The transmembrane protein BST-2 potently acts by tethering to HIV particles present on the surface of virus-producing cells. This restriction factor is very peculiar in that, unlike the other three factors that specifically inhibit retroviral infections, BST-2 displays broad-spectrum activity against a variety of enveloped viruses as well as retroviruses, e.g., Marburg virus^17^, Lassa virus^17,18^, Ebola virus^19,20^, Sendai virus^21^, influenza virus^22,23^, herpes simplex virus 1^24^, Kaposi’s sarcoma-associated herpesvirus^25^, vesicular stomatitis virus^26^, chikungunya virus^27^, SARS corona virus^28^, hepatitis B virus^29^, and hepatitis C virus^30^, human parainfluenza virus^31^, almost all of which harbor different viral antagonists against BST-2. This suggests the importance of counteracting this restriction factor for efficient viral replication. Because several *in vivo* studies have implied that the stoichiometric balance between restriction factors and viral antagonists might determine disease progression^32–35^, targeting BST-2 to enhance its endogenous expression may provide new therapeutic strategies.

Although the use of type-I interferon (IFN) may be beneficial for enhancing the expression of restriction factors including BST-2, it may be desirable to specifically upregulate target gene expression in order to avoid IFN-associated adverse effects *in vivo*. In this study, we employed newly established molecular methods using a catalytically inactive version of the clustered, regularly interspaced, short palindromic repeats (CRISPR)-associated protein 9 (Cas9)-based RNA-guided activation system^36^. This modified CRISPR-Cas9 system allows us to specifically target and activate an endogenous promoter of a gene of interest, with a nuclease-deficient mutant of Cas9 (dCas9) that binds to a single guide RNA (sgRNA) by recruiting transcription factor complex(es). By using this system, we herein attempt to enhance the endogenous expression of BST-2 to observe whether the CRISPR-based pinpoint activation of BST-2 expression is able to confer cells with inhibitory activity on HIV-1 virion production, even in the presence of the viral antagonist Vpu that counteracts BST-2 by binding and downregulating the restriction factor^37^.

## Results

**Figure 1:**
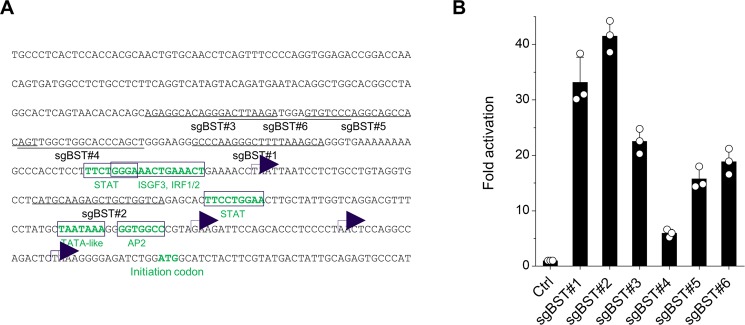
sgRNA targeting of BST-2 promoter. (**A**) The predicted BST-2 promoter sequence. Putative transcription start sites and transcription factor binding sites are shown using arrows and open boxes, respectively. The six different sgRNA target sequences are underlined. (**B**) Luciferase reporter activation by sgRNAs targeting BST-2 promoter. HeLa cells were transfected with plasmids encoding BST-2 promoter, dCas9-VP64, sgRNA, and MS2-p65-HSF1. Cells were lysed at 24 h and subjected to luciferase assays. Representative data from four independent experiments are presented as fold activation in luciferase activity compared with that in control HeLa cells (mean ± s.d., *n* = 3 technical replicates).

In a CRISPR-based RNA-guided activation system, the sgRNA targets, which are 20 nucleotides in addition to the PAM sequence (NGG), should be located within 200 base pairs of the transcription start sites of the promoter^36^, which are dispersed in the BST-2 promoter region^38^. We selected six different sgRNA targets against the BST-2 promoter (sgBST2#1–6) **(**Fig. 1A**)**. To identify the best sgRNA target sequences in the BST-2 promoter, we cloned its promoter into a firefly luciferase reporter plasmid and cotransfected HeLa cells with this promoter-indicator plasmid together with plasmids expressing sgRNA appended to the phage MS2 RNA stem loop (sgRNA-MS2 loop), as well as dCas9 fused to the herpes simplex virus transcription factor VP16 minimal activation domain termed VP64 (dCas9-VP64), and the transcription factor fusion protein that is the NF-kB trans-activating subunit p65 with the activation domain from the human heat-shock factor 1 fused to the phage MS2 coat protein (MS2-p65-HSF1), and then we performed luciferase assays. The BST-2 promoter was activated by the CRISPR-dCas9 system using all six sgRNA targets, among which sgBST2#1 and #2 revealed a 30 to 40-fold activation (Fig. 1B**)**. We therefore selected these two sgRNA target sequences for further experiments.

**Figure 2:**
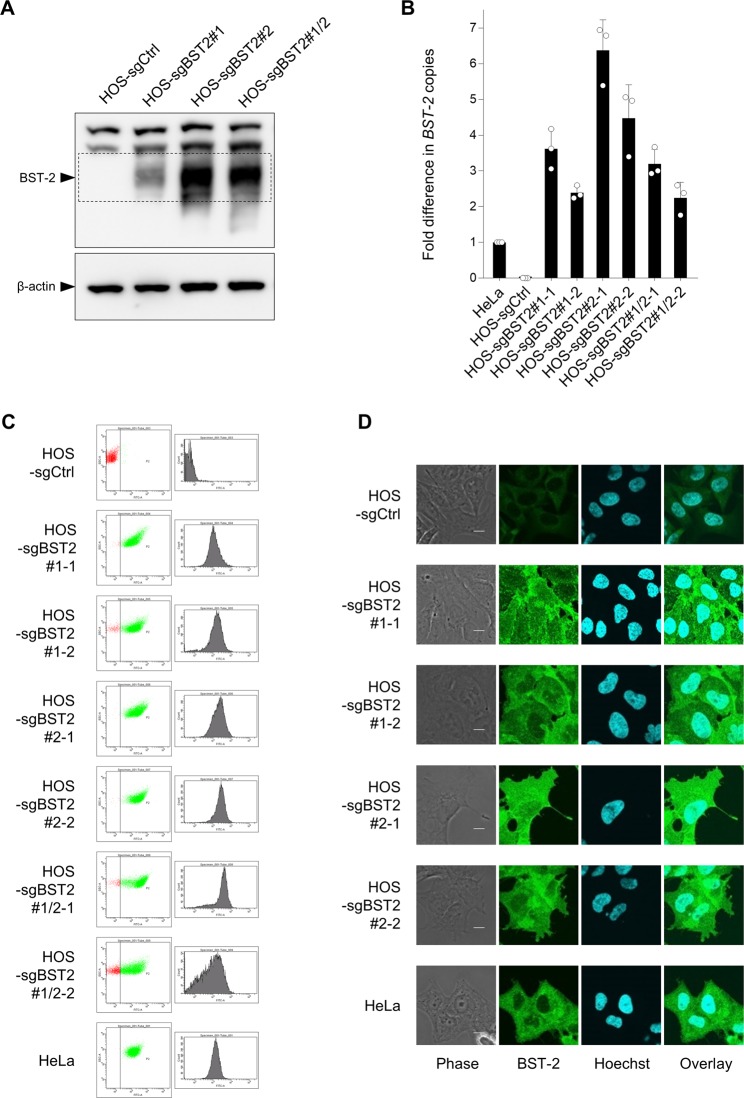
Activated expression of endogenous BST-2 by lentiviral CRISPR transduction. (**A**) HOS cells were cotransduced with lentiviruses expressing dCas9-VP64, MS2-p65-HSF1, and either BST-2-targeting sgRNA (sgBST2) #1, #2, or the combination of #1 and #2 (sgBST2#1, sgBST2#2, or sgBST2#1/2, respectively). Cell extracts derived from transduced HOS cells were subjected to immunoblot analyses using an anti-BST-2 polyclonal antibody. β-actin was used as a loading control. (**B**) HOS cells transduced in **A** were cloned (designated HOS-sgBST2#x-x) and RNAs extracted from resultant cells were analyzed by real-time RT-PCR. Data were normalized to those of the housekeeping gene *RPL27* mRNA and are shown as a fold difference in *BST-2* copies compared with those in HeLa cells (mean ± s.d. from three independent experiments). (**C**,**D**) Control and cloned HOS cells together with HeLa cells were analyzed for cell-surface expression of BST-2 by flow cytometry (**C**) or for its intracellular expression by immunofluorescence (**D**; bars, 10 μm) using anti-BST-2 polyclonal antibodies.

To examine whether these sgRNAs are indeed able to activate the endogenous expression of BST-2, we employed lentiviral vector systems for transduction. We first produced lentiviruses expressing dCas9-VP64 fusion proteins in HEK293T cells, next infected BST-2-negative HOS cells with the lentiviruses, and then selected the transduced cells with blasticidin whose resistance gene was expressed using the dCas9-VP64 vector. Next, we produced another lentivirus expressing the MS2-p65-HSF1 transcription factor fusion protein, transduced the blasticidin-selected cells with the viral vector, and selected the cells using hygromycin. Lastly, we generated a lentivirus expressing sgBST2#1 and/or sgBST2#2, and transduced the dually selected HOS cells with the viral vector and selected the cells with zeocin. By performing Western analysis, we successfully confirmed the activated expression of endogenous BST-2 protein in a polyclonal population of CRISPR-transduced HOS cells, although additive effect of two sgRNAs was not observed (Fig. 2A). Then, we cloned the transduced cells using limiting dilution and subjected the resultant single cell clone to real-time RT-PCR, flow cytometry, and immunofluorescence. Real-time RT-PCR showed that endogenous expression of *BST-2* mRNA was drastically activated in all single cell clones (Fig. 2B). Cell-surface BST-2 expression analyzed by flow cytometry was robustly increased in the cloned cells (Fig. 2C). Additionally, immunofluorescence revealed a high level of **intracellular expression** of BST-2 in the same cells (Fig. 2D). We conclude that this CRISPR-based system effectively activates BST-2 expression.

**Figure 3:**
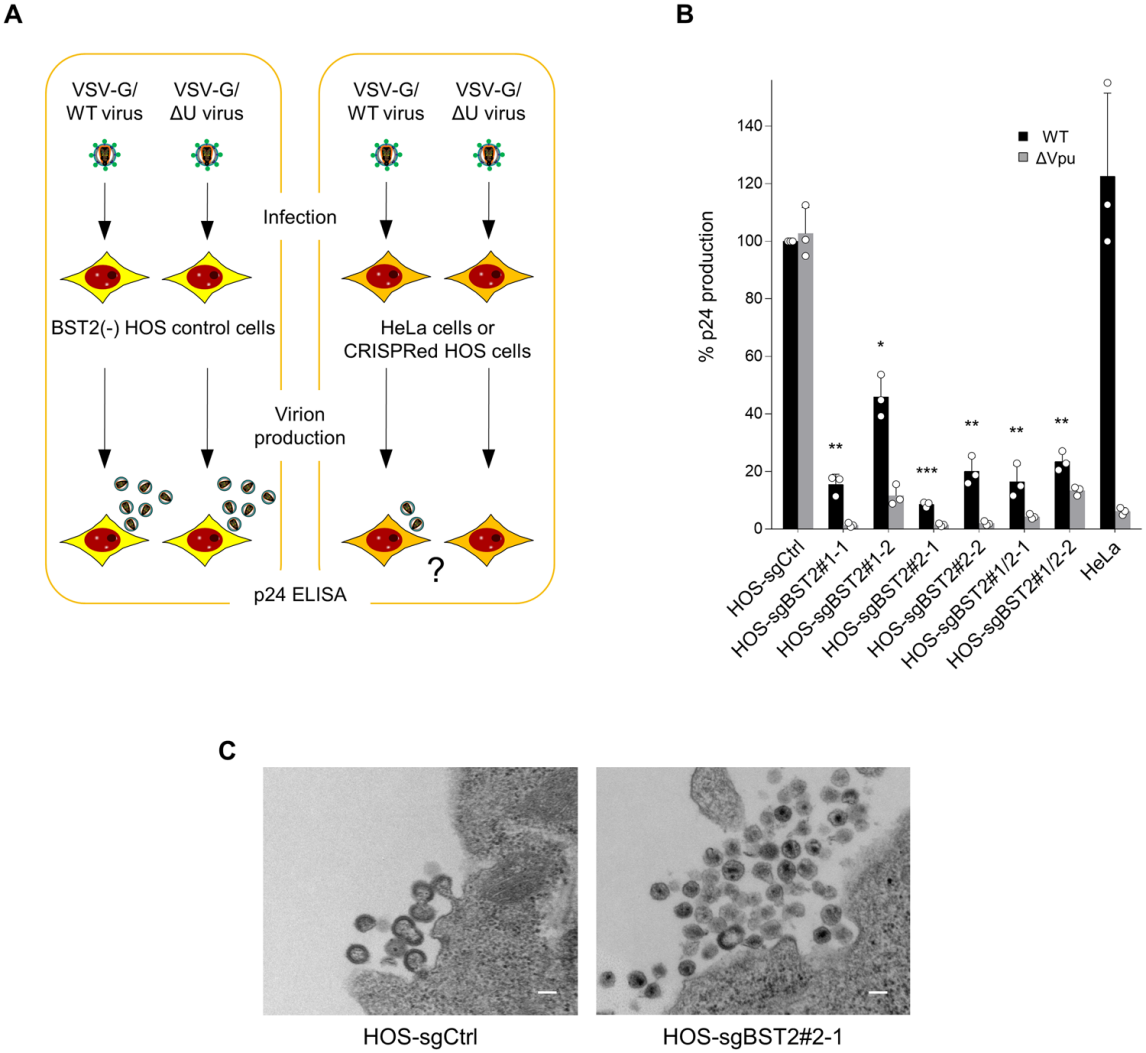
Inhibition of wild type HIV-1 production in CRISPR-transduced cells highly expressing BST-2. (**A**) Schematic flowchart of the experimental procedure for infection-based HIV-1 virion production assays. (**B**) Virion production from control and cloned HOS cells together with HeLa cells infected with Vpu-positive or -negative HIV-1 pseudotyped with VSV-G. Data are shown as a percentage of the wild-type virion production from control HOS cells (mean ± s.d. from three independent experiments) *P < 0.01, **P < 0.001, and ***P < 0.0001, compared with the wild-type viruses in control HOS cells using unpaired two-tailed Student’s *t*-test (only the wild-type data are compared for clarity). (**C**) A representative transmission electron microscopy image of wild-type HIV-1 virions accumulated at the surface of either control cells or CRISPR-transduced HOS cells highly expressing BST-2. Note that many mature virus particles after budding are accumulated at the surface of BST-2 positive cells (right), whereas typical immature virions are about to pinch off from the control cell surface (left). Bar, 0.1 μm.

We next performed infection-based virus production assays. HeLa cells or CRISPR-modified HOS cells, as well as BST-2(−) control HOS cells, were infected with either Vpu-intact or deficient VSV-G-pseudotyped viruses prepared from HEK293T cells transfected with the corresponding plasmids, and viruses produced from the infected HeLa or HOS cells were subjected to HIV-1 p24 ELISA to determine the levels of virus production (Fig. 3A). Production of not only Vpu mutant viruses but also Vpu-intact viruses were effectively inhibited in all single clone cells (Fig. 3B). Importantly, electron microscopic analyses showed that Vpu-intact viruses were indeed accumulated at the surface of BST-2 positive HOS cells (Fig. 3C).

**Figure 4:**
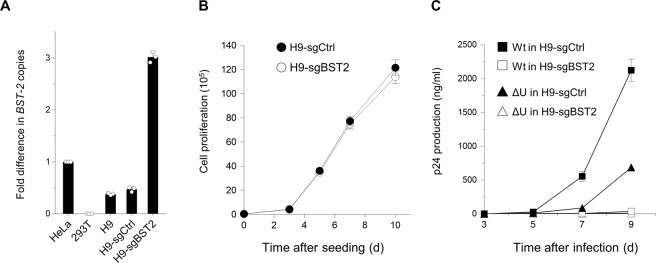
CRISPR-mediated enhancement of BST-2 expression leads to marked decrease in wild-type HIV-1 replication. (**A**) CD4-positive H9 cells with an intermediate level of *BST-2* expression were transduced with lentiviral CRISPR activation vector expressing an empty sgRNA (H9-sgCtrl) or an sgRNA targeting BST-2 promoter (H9-sgBST2), the latter of which were cloned. Cells were analyzed by real-time RT-PCR for *BST-2* expression. Representative data from three independent experiments are shown as a fold difference in *BST-2* copies compared with those in HeLa cells (mean ± s.d., *n* = 3 technical replicates). (**B**) Cell proliferation of CRISPR-transduced H9 cells (H9-sgCtrl, closed circles; H9-sgBST2, open circles). The assay was started with 20,000 cells. Data shown are representative of three independent experiments. (**C**) Virus replication in H9-sgCtrl (closed symbols) or H9-sgBST2 (open symbols). Cells (1 × 10^5^) were infected with 0.1 ng of p24 antigen of either wild-type (squares) or Vpu-defective (triangles) NL4-3 viruses. Supernatants were harvested at two-to-three-day intervals, and virus replication was monitored by p24 ELISA. Data shown are representative of four independent experiments.

Finally, we transduced the CD4-positive T-cell line H9, which exhibit an intermediate level of expression of BST-2, using the lentiviral CRISPR system to enhance endogenous expression of this protein. Real-time RT PCR showed that CRISPR-transduced H9 clonal cells indeed expressed high levels of *BST-2* mRNA (Fig. 4A) without affecting cell proliferation (Fig. 4B). We then performed viral replication assays using either wild-type HIV or Vpu-defective viruses. Consistent with the results obtained in the production assays, replication of not only the Vpu mutant but also the wild-type viruses were markedly decreased in BST-2 positive H9 cells (Fig. 4C). We therefore conclude that enhancement of endogenous BST-2 expression leads to inhibition of HIV-1 production as well as multiple-round replication, even in the presence of the BST-2 antagonist Vpu.

## Discussion

In this study, we first identified the sgRNA target sequences at the promoter regions that can activate expression of BST-2, then transduced cells with lentiviruses expressing the sgRNAs plus dCas9 together with accessory transcriptional activators and induced enhanced expression of BST-2 at both the mRNA and protein levels, resulting in successful inhibition of viral production and replication. In many cases, host restriction factors that are able to inhibit HIV infection are normally counteracted by HIV accessory proteins that act as viral antagonists. It has however been shown that the outcome of the battle between the host restriction factors and viral antagonists may be determined not only by their intrinsic activities but also by their stoichiometric balance, since overexpression of restriction factors can relieve the blockade imposed by viral antagonists^12,15,16,39–42^. This is also suggested by several studies showing that the levels of expression of restriction factors are linked to disease progression after viral infections^32–35^. In other words, even in the presence of virus-encoded antagonists, the more host restriction factors are expressed, the higher the chances to inactivate the viruses. Although type-I IFN can upregulate hundreds of IFN-stimulated genes that include many host restriction factors, the study of SIV-infected rhesus macaques showed that treatment with IFNα is effective *in vivo* only during the acute phase of infection and rather detrimental during the chronic stage due to the enhancement of systemic inflammation^43^. Even in human studies, type-I IFNs have been reported to upregulate CCR5^44^ that provides target cells for HIV-1, potentially leading to loss of CD4+ T-cells^45–47^. In this sense, it is likely that pinpoint activation of restriction factors without using IFNs might be beneficial to hosts. Indeed, our present study showed that endogenous BST-2 expression enhanced by CRISPR methods led to remarkably decreased production and multiple-round replication of HIV-1 harboring the viral antagonist Vpu, although in the case of *APOBEC3G* gene activation, its inhibitory effect on wild-type HIV-1 infection was weak^48^. Higher expression of BST-2 has been implicated in the growth and progression of cancers due to its ability to promote cell-to cell interactions and to activate NF-kB-mediated signal transduction pathways^49^. In this case, it may be better to avoid traditional overexpression systems using exogenous transgenes that lead to uncontrollably robust expression of target proteins, while CRISPR activation results in endogenously maximized levels of expression, reflecting a more natural mechanisms of action with physiologically relevant phenotypes *in vivo*^50,51^. Also, instead of the lentiviral systems that we used in this study, adeno-associated viral vectors should be applied to the future experiments to readily prepare high-titer virus stocks and to transduce primary cells. Moreover, it may be necessary to achieve specific gene delivery of CRISPR-based vectors to the infected cells. Targeting restriction factors to enhance their endogenous expression may provide a new therapeutic strategy to combat HIV infections. By using this system, we will pursue the possibility that the CRISPR-based activation of antiviral proteins, especially in the case of BST-2, could be one of the novel therapeutic strategies for inhibiting infections and preventing of transmission with HIV-1 as well as a variety of enveloped viruses.

## Methods

### DNA constructs

The luciferase-reporter promoter construct pGL4-BST2pro was created by PCR-amplifying the BST-2 promoter sequence (SwitchGear Genomics library) using DNA extracted from HOS cells and by cloning its *Nhe*I/*Xho*I-digested fragment into pGL4.10[*luc2*] (Promega). Lentiviral plasmids, lenti dCAS-VP64_Blast (expressing dCas9 fused to the VP64 transactivation domain), lenti sgRNA(MS2)_zeo backbone (expressing sgRNA linked to the MS2 bacteriophage coat protein-binding stem loop), and lenti MS2-P65-HSF1_Hygro (expressing MS2 fused to the NF-kB trans-activating subunit p65 with the activation domain from human heat-shock factor 1 (HSF1))^36^ were obtained from F. Zhang, Massachusetts Institute of Technology, through Addgene (plasmid ID 61425, 61426, and 61427, respectively). Based on an online tool CHOPCHOP ver.2^52^ that can detect the number of mismatches, specific sgRNA target sequences against BST-2 promoter were selected within 200 base pairs of the transcription start site previously mapped^38,53^ by avoiding potential off-target sites with up to two mismatches (note that CRISPR-mediated control of promoter activity is highly sensitive to even a single mismatch, probably due to a narrow window of target sequence^54,55^). Six different oligonucleotides encoding the selected sgRNAs were individually inserted into the lenti sgRNA(MS2)_zeo backbone digested with *BsmB*I to generate the lentiviral sgRNA expression plasmids pLV-sgR-BST2pro-Zeo. The lentiviral packaging vector psPAX2, a vesicular stomatitis virus G-glycoprotein (VSV-G) expression plasmid pC-VSVg, HIV-1 Rev expression plasmid pCa-Rev, HIV-1 Tat expression plasmid pLTR-Tat, the HIV-1 proviral construct pNL4-3, its envelope glycoprotein (Env)-deficient HIV-1 proviral construct pNL-E(−), and Env/Vpu-deficient construct pNL-E(−)U(−), pCAGGS mammalian expression plasmid have previously been described elsewhere^56,57^. The replication-competent Vpu-deficient HIV-1 construct pNL-U(−) was created by introducing a *Hpa*I site at the *vpu* initiation codon of pNL4-3 using QuikChange site-directed mutagenesis (Stratagene).

### Cell maintenance

HEK293T, HeLa, HOS cells were maintained in DMEM (Life Technologies) supplemented with 10% heat-inactivated FBS (Sigma). H9 human CD4-positive T-cells were maintained in RPMI (Life Technologies) supplemented with 10% heat-inactivated FBS.

### Promoter assays

HeLa cells (7 × 10^4^) were cotransfected in triplicate in a 96-well plate with 50 ng each of pGL4-BST2pro, lenti dCAS-VP64_Blast, lenti MS2-P65-HSF1_Hygro, and 100 ng of either pLV-sgR-BST2pro-Zeo or an empty vector control using the FuGENE6 transfection reagent (Promega) according to the manufacturer’s instructions. After 48 h, the cells were lysed in 100 μl of One-Glo Luciferase Assay Reagent (Promega). The firefly luciferase activity was determined with a Centro LB960 (Berthold) luminometer.

### CRISPR lentiviral transduction

HEK293T cells (5 × 10^5^) were cotransfected with 40 ng of pC-VSVg, 1 μg of psPAX2, and 1 μg of either lenti dCAS-VP64_Blast, lenti MS2-P65-HSF1_Hygro, or pLV-sgR-BST2pro-Zeo (#1 or #2), using the FuGENE6 transfection reagent (Promega) according to the manufacturer’s instructions. After 48 h, the supernatants were treated with 37.5 U ml^−1^ DNase I (Roche Applied Science) for 37 °C for 30 min and then harvested, and the amount of p24 antigen was measured using an HIV-1 p24-antigen capture enzyme-linked immunosorbent assay (ELISA) kit (XpressBio). BST-2-negative HOS cells (4 × 10^5^) or H9 cells (1 × 10^5^) that lowly express BST-2, were transduced with 300 ng of each of the dCas9-VP64-expressing lentivirus and the MS2-p65-HSF1-expressing lentivirus. After 48 h, the transduced cells were cultured in the presence of blasticidin (10 μg ml^−1^, Life Technologies) and hygromycin (300 μg ml^−1^, Thermo Fisher) and were selected for 7 days. Cells stably expressing dCas9-VP64 together with MS2-p65-HSF1 were further transduced with the lentivirus expressing an empty sgRNA or an sgRNA against the BST-2 promoter (transduced cells were designated H9-sgCtrl or H9-sgBST2, respectively), and selected by zeocin (200 μg ml^−1^, InvivoGen) for another 7 days. The resultant stable H9-sgBST2 cells were subsequently cloned by limiting dilution.

### Real-time RT-PCR

To measure endogenous and transduced levels of *BST-2* expression, total RNA was extracted from HeLa cells, HOS (control or transduced) cells, and H9 cells (control or transduced) using a ReliaPrep RNA Cell Miniprep system (Promega). Real-time RT-PCR was performed with Mx3000P (Stratagene) using the QuantiTect Multiplex RT-PCR (Qiagen) according to the manufacturer’s instructions. The specific primers and probes used were as follows: *BST-2*, 5′-GAG CTT GAG GGA GAG ATC ACT AC-3′/5′-ATT CTC ACG CTT AAG ACC TGG TT-3′/HEX-5′-TCT CTT CTC AGT CGC TCC ACC TCT GC-3′-black-hole quencher 1 (BHQ1); ribosomal protein L27 (RPL27), 5′-CAA TCA CCT AAT GCC CAC AAG-3′/5′-TTC TTG CCT GTC TTG TAT CTC TC-3′/Cy5-5′-TCA AAC TTG ACC TTG GCC TCC CG-3′-BHQ2. *BST-2* mRNA levels were normalized with *RPL27* mRNA levels.

### Immunoblotting assays

Cells were lysed in 500 μl of lysis buffer (20 mM Tris, pH 7.4, 200 mM NaCl, 1 mM EDTA, 2.5% n-octyl-β-D-glucoside, and Complete Protease Inhibitor Mixture (Roche Applied Science)). Cell extracts were then subjected to gel electrophoresis and transferred to a nitrocellulose membrane. The membranes were probed with an anti-BST-2 mouse polyclonal antibody (Abnova). Reacted proteins were visualized by chemiluminescence using an ECL Western blotting detection system (GE Healthcare) and monitored using a LAS-3000 imaging system (Fujifilm).

### Flow cytometry

HeLa or HOS cells were incubated with an anti-BST-2 polyclonal antibody^58^, followed by staining for 30 min on ice with an anti-rabbit IgG conjugated with Alexa 488 (Molecular Probes). Cells were then washed extensively with PBS plus 4% FBS and fixed with 4% formaldehyde in PBS. Cells were then analyzed by flow cytometry using BD FACS Canto II, and the data were collected and analyzed with the BD FACS Diva Software.

### Immunofluorescence microscopy

HeLa or HOS cells were collagen-coated plated on 13-mm glass coverslips, and cultured for 3 h before fixation. The fixed cells were permeabilized with 0.05% saponin for 10 min and immunostained with the anti-BST-2 polyclonal antibody^56^. Secondary anti-rabbit IgG conjugated with Alexa 488 (Molecular Probes) was used at 5 μg ml^−1^. DNA staining with Hoechst (Molecular Probes) was performed at 0.5 μg ml^−1^. All immunofluorescence images were observed using Fluoview FV10i (Olympus).

### Virion production assays and transmission electron microscopy

HEK293T cells (5 × 10^5^) were cotransfected with 1 μg of the proviral construct pNL-E(−), or pNL-E(−)U(−), together with 40 ng of pC-VSVg and 1 μg of the empty pCAGGS by using FuGENE 6. After 48 h the supernatants were harvested and subjected to p24-antigen capture ELISA. CRISPR-transduced HOS cells seeded at 5 × 10^4^ cells were infected with 10 ng each of VSV-G-pseudotyped HIV-1. Sixteen hours later the cells were washed with PBS and 1 ml of fresh complete medium was added. For virion production assays, supernatants were harvested after 24 h and subjected to HIV-1 p24-antigen capture ELISA. For transmission electron microscopy, infected cells were harvested after 24 h with a cell scraper and washed twice with ice-cold PBS. Cells were then prefixed with 2.5% glutaraldehyde and 2% paraformaldehyde in 0.1 M phosphate buffer, pH 7.4, for 2 h at room temperature, postfixed in 1% osmium tetroxide, and embedded in Epon 812 (TAAB Laboratories). Ultrathin sections were stained with uranyl acetate and lead citrate and then observed under a transmission electron microscope (HT7700; Hitachi) at 80 kV.

### Replication assays

HEK293T cells (5 × 10^5^) were cotransfected with 2 μg of the proviral construct pNL4-3, or pNL- U(−) by using FuGENE 6. After 48 h the supernatants were harvested and subjected to p24-antigen capture ELISA. H9-sgCtrl and H9-sgBST2 cells (1 × 10^5^) were infected for 3 h with either wild-type or Vpu-defective viruses (0.1 ng of p24 antigen), washed extensively with serum-free medium and then cultured in fresh complete medium. Supernatants were sampled every 2 to 3 d, and p24 antigen production was quantified using ELISA.

### Statistical analyses

Values are presented as the mean ± s.d. for three or four independent experiments, determined on the basis of pilot experiments to estimate the effective numbers. Statistical comparisons were made using a paired two-tailed Student’s *t*-test, and a *P* < 0.05 was considered statistically significant.

## Supplementary information

Supplementary Figure 1
