# Supplementary material for: CRISPR-mediated activation of endogenous BST-2/tetherin expression inhibits wild-type HIV-1 production
Source: Sci Rep. 2019 Feb 28;9:3134. doi: 10.1038/s41598-019-40003-z (PMC6395588; doi:10.1038/s41598-019-40003-z)
Supplement: Supplementary file 1 — Supplementary Figure 1 [file 41598_2019_40003_MOESM1_ESM.pdf]

Supplementary information

CRISPR-mediated activation of endogenous BST-2/tetherin expression inhibits wild-type HIV-1 production

Yanzhao Zhang, Seiya Ozono, Weitong Yao, Minoru Tobiume, Shoji Yamaoka, Satoshi Kishigami, Hideaki Fujita, and Kenzo Tokunaga

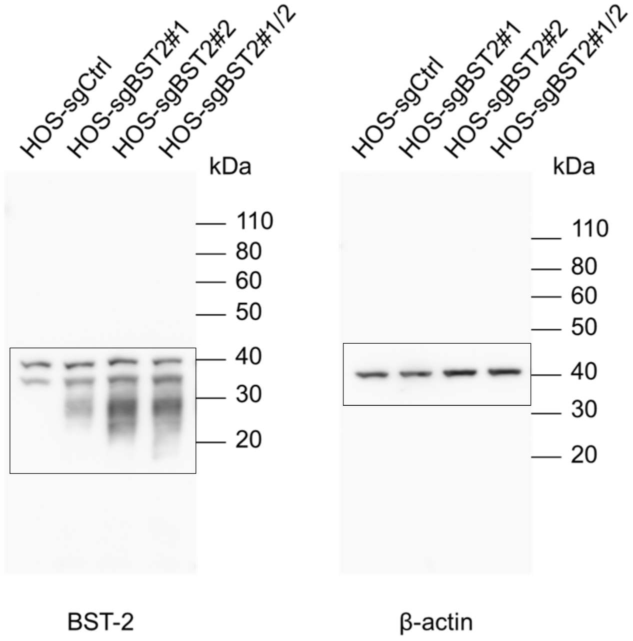

Supplementary Figure 1.

Original uncropped images of Western Blot in Figure 2A. The PVDF membrane was incubated with an anti-BST-2 antibody, then stripped and reprobed with an anti-β-actin antibody for a loading control. Images shown in Fig. 2A were cropped from the boxed areas, and the brightness/contrast was adjusted equally across the entire image using Photoshop CS6.
